# Salinity stratification controlled productivity variation over 300 ky in the Bay of Bengal

**DOI:** 10.1038/s41598-017-14781-3

**Published:** 2017-10-31

**Authors:** R. Da Silva, A. Mazumdar, T. Mapder, A. Peketi, R. K. Joshi, A. Shaji, P. Mahalakshmi, B. Sawant, B. G. Naik, M. A. Carvalho, S. K. Molletti

**Affiliations:** 1 CSIR, National Institute of Oceanography, Donapaula, Goa, 403004 India; 2 ACEMS, Queensland University of Technology, Brisbane, QLD4000 Australia; 3 Geological Survey of India, Salt lake, Kolkata, 700091 India; 4 Centre for Marine Living Resources & Ecology, Kochi, 682037 Kerala India; 5 Flat No. CS-1, Block-C, Astral Garden, Panaji, 403004 Goa India; 6 Delta Studies Institute, Andhra University, Visakhapatnam, 530017 Andhra Pradesh India

## Abstract

The unique hydrographic setting of the Bay of Bengal (BoB) makes it an ideal tropical marine system to study the influence of regional and global forcings on productivity and [CO_2aq_] through the late quaternary. Enormous fresh water flux into the BoB and consequent salinity stratification significantly weaken the convective mixing and wind driven processes which are commonly responsible for transport of nutrients to the euphotic zone driving primary productivity. Here we present a high resolution organic carbon-CaCO_3_ MAR and δ^13^C_TOC_ records for the last 300 ky from the BoB. The results show significant productivity variation at marine isotope sub-stages and millennial timescales. Colder sub-stages and stadials (Dansgard-Oeschger cycle) show a boost in productivity which may be attributed to thinning of low salinity cap, thereby facilitating efficient nutrient transport across the euphotic zone by the combination of wind driven processes (entrainment and upwelling), convective mixing and cold core eddies. The [CO_2aq_] was a net result of global pCO_2_ variation and regional processes. Our long term high-resolution data indicates a possibility of marked change in productivity/biogeochemistry of BOB in the future due to global warming, thus affecting the coastal economy.

## Introduction

The Bay of Bengal, a tropical semi-enclosed basin in the northern Indian Ocean is the largest bay in the world, bordered by India, Sri Lanka, Bangladesh, Myanmar and the Andaman-Nicobar Islands. Ganga-Brahmaputra (G-B), Irrawaddy, Godavari, Mahanadi, Krishna and Kaveri rivers contribute 60% of the total freshwater received by the BoB of which the G-B river system contributes^1^ 44%. Enormous riverine water flux (2.95 × 10^12^m^3^/yr)^1^ and excess of precipitation over evaporation results in a stable water column (50–80 m) salinity stratification^2^ in BoB, in contrast to other Indian Ocean regions. The surface salinity in BoB is lowest (~29 psu) above 20°N latitude and increases to 34 psu around 7°N latitude. The salinity gradient^2^ from north to south decreases from 5.5 to 1. The salinity stratification results in a shallow mixed layer depth (MLD ~ 5 to 30 m) which shows marked regional and seasonal range^3^. The salinity stratification enhances the stability of the water column and prevents mixing with the underlying cooler waters leading to high sea surface temperatures (~28 °C) throughout the BoB^4^. The water column stability does not allow the prevailing wind (5–10 ms^−1^)^5^ to disrupt the stratification^6^ significantly except during cyclonic episodes. The salinity stratification affects the vertical distribution of heat in the near surface layers of BoB and can influence processes such as the active–break cycles of summer monsoons and development of regional tropical cyclones^7^. Owing to these enigmatic oceanographic characteristics, physical processes like convective mixing and wind driven processes (upwelling and nutrient entrainment) which are responsible for the transport of nutrients across the euphotic zone leading to primary productivity are significantly weaker in BoB compared to that of Arabian Sea^8^. As a result, maximum productivity in the open ocean BoB is associated with subsurface chlorophyll maxima (SCM ~40 to 90 mbsl)^2,5^ in both the central and western bay throughout the year^2,9^, whereas, surface productivity in BOB is limited to the coastal regions receiving nutrients along with perennial runoff from the rivers^9^. The depth of SCM is controlled by the vertical transport of nutrient by mesoscale cold core eddies (anticyclonic)^2,5,6,9,10^. The genesis of mesoscale eddies are attributed mainly to the interaction of Kelvin wave propagating along the coastal boundary with the northward flowing western boundary current and also by the breaking of Rossby waves propagated from the eastern boundary of BoB^10^.

In view of this unique oceanographic setting and being one of the most vulnerable^11^ regions in the world due to climate change, BoB is one of the hotspots for climate change research. Thus, it is pertinent to investigate how marine productivity and surface water dissolved CO_2_ ([CO_2aq_]) responded to highly variable past climatic conditions. Understanding the natural variability of the paleoceanic proxies at high temporal resolution will allow more accurate modeling of the consequences of future warming.

### Study area

**Figure 1:**
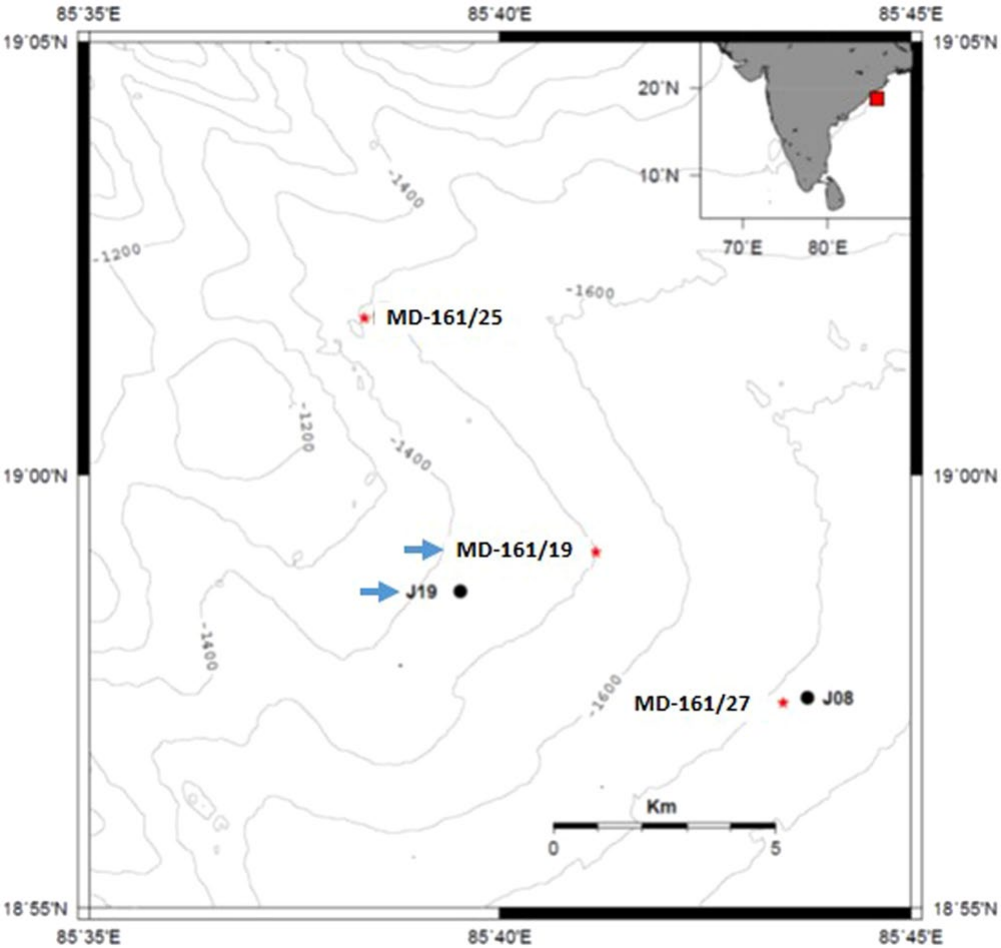
Location of cores MD161–19 and NGHP-19a (J 19) on the bathymetry map. The two core locations are indicated by thick blue arrows. The map is prepared using Generic Mapping tools software (GMT, version 4.5.1.15; www.gmt.soest.hawaii.edu/gmt4).

As part of this investigation, a giant Calypso piston corer was used on board ORV *Marion Dufresne* (MD-161) for the retrieval of the sediment core MD161-19 (core length: 39 m) off Mahanadi Basin (Lat.:18°59.1020; Long.:85°41′′.1669′′) in western BoB at a water depth of 1480 m (Fig. 1).

## Results

**Figure 2:**
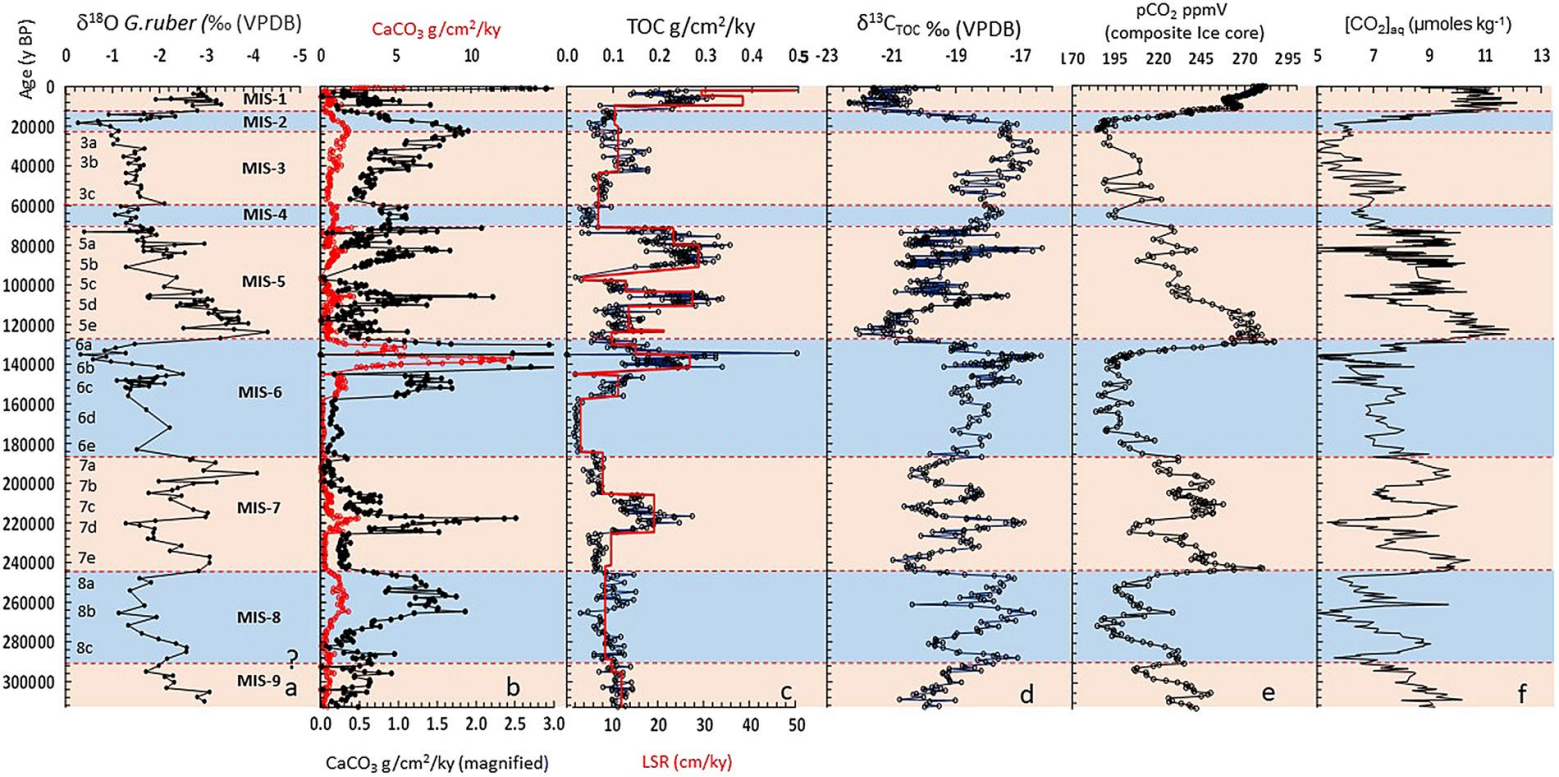
Geochemical profile through core MD161–19. Blue sections indicate cold MIS and orange sections indicate warm MIS. (**a)** δ^18^O_*G. ruber*_ profile indicating marine isotope stage and sub-stages. Marine isotope stage (MIS) boundaries (1 to 8) and substages^13^ are demarcated by dashed lines. (**b**) CaCO_3_ MAR. (**c)** TOC MAR and LSR. (**d)** δ^13^C_TOC_ (‰VPDB). (**e)** pCO_2_ ppmv (composite Ice core) (**f**) Temporal variation [CO_2aq_].

The age-depth model (Supplementary Fig. 1 and Supplementary Table 1) for MD161–19 is based on the calibrated radiocarbon ages^12^ and correlation of our δ^18^O_G.ruber_ profile with the standard δ^18^O profile^13^. Eight marine isotope stages (MIS 1-8) representing glacial and interglacial changes are demarcated in Fig. 2. The linear sedimentation rate (LSR) varies from 1.5 to 38.6 cm/ky except in the last 1114 yr where the sedimentation rate reaches a peak value of 241 cm/ky. Total inorganic carbon (TIC) concentrations range from 0.01% to 5.6 wt% (Supplementary Table 2). The CaCO_3_ mass accumulation rate (MAR) reaches a peak value of 12.6 g/cm^2^/ky within the time window of 126885–145000 yr. Beyond this time window, the CaCO_3_ MAR ranges from 0.02 to 0.26 g/cm^2^/ky except during the period of 689–2000 yr where the MAR reaches a maximum of 0.66 g/cm^2^/ky. The Total organic carbon (TOC) value ranges from 0.15% to 2.9 wt% (Supplementary Table 2). The TOC MAR ranges from 0.01 to 0.5 g/cm^2^/ky barring the high flux of 2.0 g/cm^2^/ky within 689–2000 y. The δ^13^C_TOC_ values range from −22.3 to −16.3‰ (VPDB).

## Discussion

CaCO_3_ and TOC MARs (Fig. 2b,c) are considered here as proxies for past productivity variations in response to changes in regional forcing like surface water hydrography and nutrient supply in the BoB. The MAR deducts the siliciclastic dilution effect and indicates CaCO3 and TOC fluxes on the sea floor assuming no loss or gain of material in the sediment^14,15^. The colder sub-stages^13^ (Fig. 2a) are associated with enhanced CaCO_3_ MAR, whereas, warmer sub-stages allied with diminished CaCO_3_ MAR. We attribute this marked temporal variations in CaCO_3_ MAR primarily to changes in calcite productivity^16,17^ rather than differential preservation^18^. The core MD161–19 collected at a water depth of 1480 mbsl, is well above the reported depth of calcite saturation/lysocline (~3000 m) in the BoB^19^. Comparison of shallow (730–809 m) and deep (1727–2250 m) water CaCO_3_ particle flux data^20^ from northern BoB (NBBT) show minor differences, suggesting lack of major calcite dissolution at water depths of ~2000 m. Based on foraminifera distribution in the surface sediments of BoB, ~2000 mbsl was suggested as the calcite lysocline depth^21^. Since, pre-industrial pCO_2_ was at least 100 ppmv less than the present day value, lysocline depth was definitely deeper than the present. Post depositional dissolution of calcite by anaerobic biogeochemical reactions like sulfate reduction is unlikely, since pore fluid chemistry data^12^ of the current core shows a drop in pore-water calcium concentrations and increase in total alkalinity (TA) with depth below the sea floor. The increase in TA is attributed to organoclastic sulfate reduction and/or anaerobic oxidation of methane (AOM) which promotes calcite precipitation rather than dissolution^22^. However, anaerobic oxidation of pyrite by Fe^3+^ ion during deep burial diagenesis may result in a drop in pH leading to some calcite dissolution. Influence of such diagenetic processes cannot lead to the observed systematic temporal variation in calcite burial flux. The apparent increase in δ^18^O_G.ruber_ values coupled with enhanced CaCO_3_ MAR and vice versa (Fig. 2a,b) and an overall positive statistical correlation between δ^18^O_G.ruber_ and CaCO_3_ MAR (Supplementary Fig. 2a and 2b and Supplementary Table 3) suggests an underlying climatic control on productivity.

Total organic carbon and CaCO_3_ MARs show an overall similarity in temporal trends (except in MIS-2 and 4) which can be attributed to carbonate productivity variation coupled with enhancement of phytoplankton biomass mainly diatoms^23,24^ in BoB^25^. Such a coeval pattern in productivity may be attributed to nutrient availability. The TOC MAR shows relatively stronger influence of sedimentation rate than CaCO_3_ MAR (Fig. 2b,c). This may be attributed to significantly lower %RSD of TOC wt% (~30.5%) compared to that of TIC wt % (105%). TOC MAR is also influenced by high sedimentation rate owing to both detrital dilution as well as enhanced preservation of organic carbon^15^.

Here we establish a link between temporal variation in regional forcing and paleo-productivity in the BoB. Studies from BoB have shown a significant reduction in monsoonal intensity/fresh water flux during colder isotope sub-stages and opposite in warmer sub-stages^26^. This observation is also supported by multiple core data from BoB^27^ suggesting enhanced riverine fresh water flux (due to increased monsoonal precipitation) in the northern BoB during early to mid-Holocene and diminished fresh water flux (reduced monsoonal precipitation) during the last glacial maxima (LGM). Overall diminished fresh water flux during colder and arid sub-stages caused thinning of low salinity cap leading to destabilization of the water column stratification which triggered enhanced nutrient entrainment by wind driven processes and convective mixing leading to enhanced productivity (enhanced CaCO_3_ MAR).

**Figure 3:**
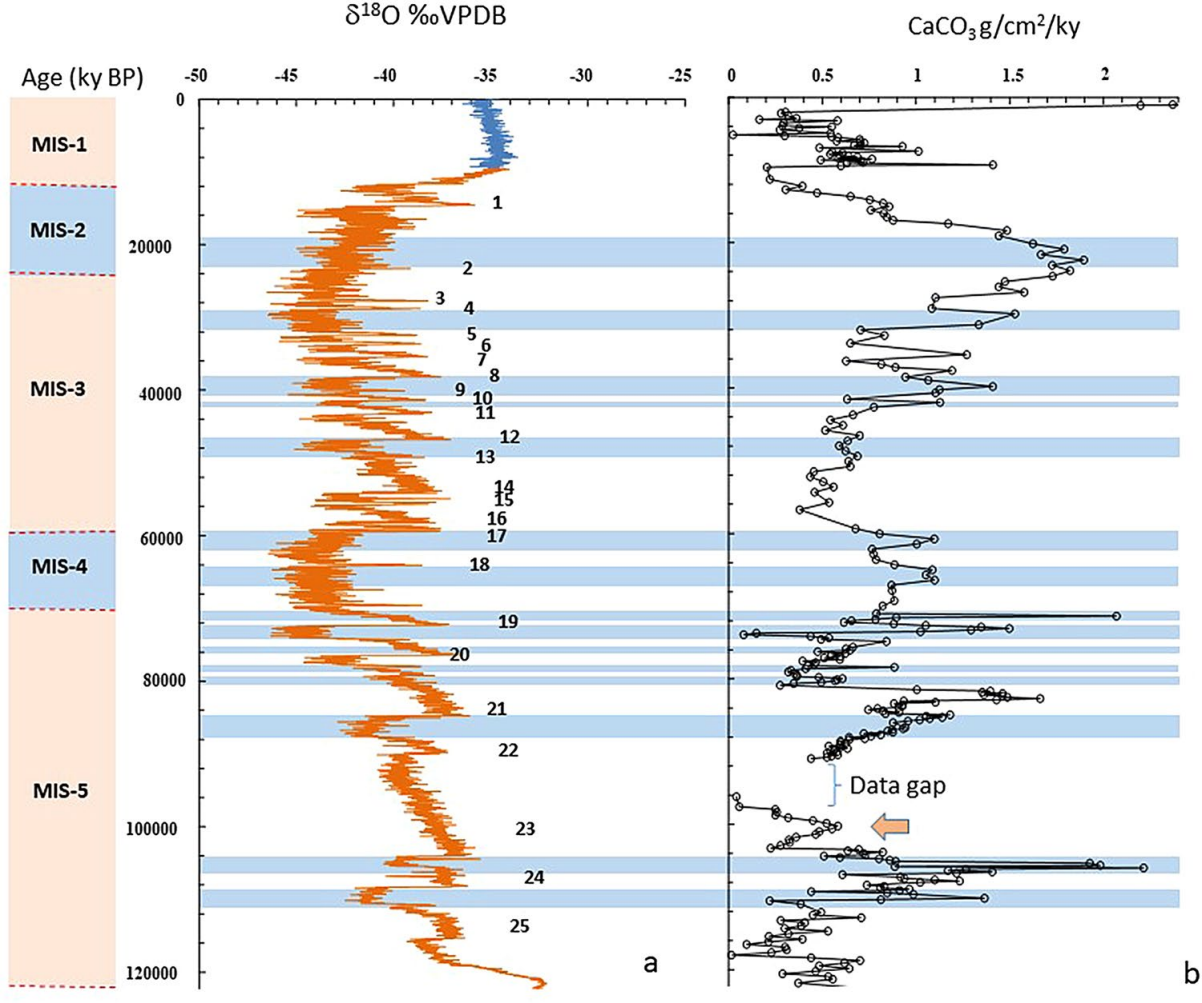
Millennial scale changes in CaCO_3_ MAR. (**a**) δ^18^O ‰VPDB profile constructed from NGRIP (North Greenland Ice Core Project) data, Daansgard-Oeschger (D-O) events are numbered from 1 to 25^13^. The blue coloured zones indicate the colder events in between the D-O events. (**b**) Temporal variation in CaCO_3_ MAR.

Exceptionally high CaCO_3_ productivity during colder sub-stage MIS 6a may be attributed to the sustained intensification of the physical forcings. Inferred^28^ intensification of NE monsoon characterized by cold dry winds and drop in river discharge during the last glacial maxima (LGM) gives further credence to our hypothesis. Cold core eddy and wind driven shoaling of nitrate into the MLD and enhanced productivity has been reported during the winter monsoon in BOB^9^. On the other hand, the marked drop in CaCO_3_ MAR during warmer and humid sub-stages may be attributed to thickening of low salinity cap and stabilization of water column (shallow MLD). A similar relationship is also apparent at the stadial/interstadial time scale (Fig. 3). The interstadials (D-O events) commonly observed at high latitudes have also been identified in the BoB^26^ and Arabian Sea^29^. The interstadials are supposed to have experienced relatively warm/ humid conditions and stronger monsoonal rainfall^26^ in contrast to the cold/ arid and weak monsoonal conditions during the stadials. The sharp drop in CaCO_3_ MAR during the interstadials and enhancement during the stadials supports the influence of freshwater flux on productivity even at millennial scale. During the warmer events, enhanced glacial melt water^30^ possibly contributed to the overall fresh water flux of the G-B river system for a short period of time.

The warmer/ humid and cold/arid time windows are also associated with relative increase and decrease in atmospheric pCO_2_ respectively (Fig. 2e). The near mirror image relation of δ^13^C_TOC_ profile with the ice core pCO_2_ record (Fig. 2d,e and Supplementary Table 2) shows the influence of atmospheric pCO_2_ on the carbon isotope ratios of particulate organic matter. A positive statistical correlation between the temporal trends of δ^13^C_TOC_ (−22.3 to −16.3‰ VPDB) and CaCO_3_ MAR (Supplementary Fig. 2a and 2b and supplementary text) indicates an overall underlying paleoclimatic control on both the parameters. The carbon isotopic composition of marine phytoplankton biomass during CO_2_ fixation via photosynthesis depends primarily on the ambient extracellular dissolved CO_2_ concentration ([CO_2aq_])^31–34^ (Fig. 2f). However, the net cellular carbon isotope fractionation depends on phytoplankton species variation^35^, solar irradiance and growth rate at seasonal scale^36^. Carbon isotope ratios of [CO_2aq_] normally range from ~ −9 to −11‰ VPDB^37^. Under [CO_2aq_] replete-conditions photosynthetic biomass show^13^C depletion, while, depletion of [CO_2aq_] leads to active-HCO_3_^−^ (δ^13^C~1‰ VPDB) ion transport inside the cell via an active inorganic carbon concentrating mechanism (CCM)^33^ and results in carbon isotopic enrichment of phytoplankton biomass^34^. The CCM pathway involves active uptake of HCO_3_^−^ ions into the cell and subsequent conversion to CO_2_ (catalyzed by the carbonic anhydrase) and fixation by RubisCO. The calculated [CO_2aq_] values (~5 to 11.6 micromoles/kg) (Fig. 1f) using the equation

1 $${([{{\rm{CO}}}_{2}]{\rm{aq}}=({{\rm{\delta }}}^{13}{{\rm{C}}}_{{\rm{TOC}}}+12.6)/-0.8)}^{31}$$

are comparable to the estimated paleo marine [CO_2aq_] record^38^. The [CO_2aq_] profile (Fig. 1f) shows a gross similarity with the atmospheric pCO_2_ profile indicating dependence of [CO_2aq_] on atmospheric pCO_2_, on the other hand, observed non-synchronous variation in [CO_2aq_] and atmospheric pCO_2_ at finer time scale may be attributed to processes including CO_2_ draw down via productivity fluctuation^39,40^ and/or influx of deep water CO_2_ through physical forcing. The calculated [CO_2aq_] values may also be affected by the presence of some terrestrial organic matter in the sediments. Variation in δ^13^C values (~0.4‰ VPDB) of ice core based atmospheric CO_2_ record^41^ is minor compared to the fluctuation in δ^13^C_TOC_ (~6‰ VPDB) reported in our work, however, this component may be considered for more precise calculation of [CO_2 aq_].

We conclude here that, in contrast to the present scenario, BoB experienced remarkable oscillations in productivity and surface water CO_2_ budget during the last 300 ky, controlled by variation in the intensity of global and regional physical processes. In view of the alarming influence of global warming on marine productivity^42^, monsoonal variability^43^ and ocean acidification^44^, the high-resolution long term natural variability observed here will be useful in vulnerability modeling of BoB^11^. The paleo productivity data coupled with δ^15^N may also help in reconstructing paleoxygenation and denitrification^45^ processes in BOB.

## Method

### Coring and sample preservation

A giant Calypso piston corer was used on board ORV *Marion Dufresne* (MD-161)^12^ for the retrieval of the sediment core MD161–19 off Mahanadi Basin at a water depth of 1480 m (Fig. 1) at Lat: 18° 59.1092′′N Long: 85° 41.1669′′E. The sea bottom temperature at this location was 4.4 °C. The core was subsampled into 5 cm thick slabs. Aliquots for organic geochemistry was preserved at 2 °C to arrest further microbial activity. Samples were freeze dried at the earliest and stored at 2 °C (dark) for hydrocarbon extraction.

### Separation of planktic foraminifera and oxygen isotope ratio measurement

200 dried and weighed aliquots of the samples of MD-161-19 were suspended in distilled water and gently sieved through a 63 µm mesh sieve. Approximately 15–20 clean tests of *Globigerinoides ruber* with the size ranging from 250 to 355 µm were picked from the oven dried > 63 µm fraction for carbon and oxygen stable isotope ratio measurement. Prior to the analyses, the foraminiferal tests were broken, cleaned in 5–10% H_2_O_2_ followed by ultra-sonication in distilled water and methanol to remove contaminants. Samples for oxygen and carbon stable isotope ratios were analyzed in a Kiel III carbonate preparation device interfaced with a Finnigan-MAT 252 isotope ratio mass spectrometer at the Department of Geological Sciences, University of Florida. This is a dual inlet system having a precision of 0.04‰ VPDB for δ^13^C and 0.08‰ VPDB for δ^18^O for calcite standard NBS-19. The results are presented in Supplementary Table 1.

### TIC, TOC contents, and δ ^13^C measurement

600 samples were desalinated and powdered prior to compositional measurements. Total Inorganic Carbon (TIC) was determined by carbon coulometer (UIC-CM5130). The accuracy of TIC content of standard reference material (Ultrapure CaCO_3_ from Sigma-Aldrich) was 12.0 ± 0.25 wt%. Total carbon (TC) content was measured by the elemental analyzer (Thermo EA1112). Total organic Carbon (TOC) content was calculated by subtracting TIC from TC. 2, 4-DNP was used as a calibration standard for TC. Reproducibility for TC in NIST-SRM1944 sediment standard was found to be 4.4 ± 0.2 wt%. Carbon isotope measurement of total organic carbon (δ^13^C_TOC_) was carried out on decarbonated sediments. A Thermo-Finnegan Delta-V-Plus continuous flow isotope ratio mass spectrometer coupled to an elemental analyzer (Thermo EA1112) was used for C isotope ratio measurements. The external standard reproducibility calculated for δ^13^C_TOC_ using IAEA-C3 cellulose standard was −24.7 ± 0.1‰ (VPDB). The results are presented in Supplementary Table 2.

### Calculation of porosity and dry bulk density (DBD)

Dry bulk density for our core was calculated using the DBD-porosity relation for we have taken the help of porosity DBD relation obtained from core number NGHP-19^46^. MD161–19 and NGHP-19 are in close proximity and have similar lithology and porosity and profile for the top 40 m. The linear equation obtained between porosity and DBD data for NGHP-19 (DBD = [(porosity−98.86)/−35.75)] with an r^2^ of 0.99 is used to calculate the DBD for MD161–19. CaCO_3_ MAR was calculated as (DBD* Sed Rate* 8.33*TIC wt%/100) and TOC MAR was calculated as (DBD* Sed Rate* TOC wt%/100). For porosity measurements, a measured volume of sediment was dried at 105 °C. Moisture content in the sediment was calculated from the difference in wet and dry weight of sediments. Porosity was calculated as (volume of sediment pore-water/wet sediment volume weight) × 100. The results are presented in Supplementary Table 2.

### Statistical analyses

Simple moving average filter: The moving average calculates the mean of the data in a particular period for a large dataset. It is simply used to reduce the random fluctuations generated in a big time series data. To calculate simple moving average (SMA), every data point is given equal weightage. The mathematical expression for estimating the SMA for a period of *n* in a time series data is as

2 $$SM{A}_{n}=\frac{1}{n}\sum _{t=k-n+1}^{k}{y}_{t}$$

where, *k* is the position of the period and *y*_*t*_ is the variable to be filtered at the time *t*^47,48^.

In Supplementary Fig. 2a, we have exhibited the raw data for CaCO_3_ MAR, δ^18^O_G.ruber_, and the δ^13^C_TOC_ with their estimated SMA filtered data for the whole time series. It is to be carefully noted that we have lesser number of available data points for δ^18^O_G.ruber_ with respect to the others. To calculate the Pearson’s correlation coefficient between CaCO_3_ MAR and δ^18^O_G.ruber_, we have truncated the CaCO_3_ MAR string as same as the size of the δ^18^O_G.ruber_ data string. As a consequence of that, we have plotted the full CaCO_3_ MAR with the reduced one. In all the cases, the filtered lines can efficiently follow the trend of the raw data after removing the fluctuations.

Pearson’s correlation coefficient: To quantify the strength of association among two simultaneously evolving quantities, there are many statistical approaches like covariance, correlation, etc. We have opted the correlation coefficient as an index here^49,50^. The Pearson’s correlation coefficient (*r*_xy_) for *n* number of data points, can be written as

3 $${r}_{xy}=\frac{{\sum }_{i=1}^{n}({x}_{i}-\bar{x})({y}_{i}-\bar{y})}{\sqrt{{\sum }_{i=1}^{n}{({x}_{i}-\bar{x})}^{2}{\sum }_{i=1}^{n}{({y}_{i}-\bar{y})}^{2}}}.$$

In the scatter plot (Supplementary Fig. 2b), we have plotted the δ^18^O_G.ruber_ and the δ^13^C_TOC_ raw data as well as the smoothened data with respect to CaCO_3_ MAR. We consider the axis of CaCO_3_ MAR in Log scale to cover the full range of CaCO_3_ MAR time series data points in a presentable form. The linear trends in the scattered points for both δ^18^O_G.ruber_ and δ^13^C_TOC_ with CaCO_3_ MAR encourages us to assess their mutual association through Pearson’s correlation coefficient (*r*_xy_). We have calculated *r*_xy_ on the full data before and after using the SMA filter. The *r*_xy_ values (Supplementary Fig. 2a) improves after removal of the local random fluctuations, but cannot affect the exact trend.

## Electronic supplementary material

Supplementary information

Supplementary Data Table-1

Supplementary Data Table-2
