# Author Correction: Salinity stratification controlled productivity variation over 300 ky in the Bay of Bengal

**DOI:** 10.1038/s41598-018-23596-9

**Published:** 2018-04-11

**Authors:** R. Da Silva, A. Mazumdar, T. Mapder, A. Peketi, R. K. Joshi, A. Shaji, P. Mahalakshmi, B. Sawant, B. G. Naik, M. A. Carvalho, S. K. Molletti

**Affiliations:** 1 CSIR, National Institute of Oceanography, Donapaula, Goa 403004 India; 2 ACEMS, Queensland University of Technology, Brisbane, QLD4000 Australia; 3 Geological Survey of India, Salt lake, Kolkata 700091 India; 4 Centre for Marine Living Resources & Ecology, Kochi, 682037 Kerala India; 5 Flat No. CS-1, Block-C, Astral Garden, Panaji, 403004 Goa India; 6 Delta Studies Institute, Andhra University, Visakhapatnam, 530017 Andhra Pradesh India

Correction to: *Scientific Reports* 10.1038/s41598-017-14781-3, published online 31 October 2017

The Authors missed out a previous study on a similar topic. The additional reference is listed below as reference 1, and should appear in the text as below.

In the Discussion section,

“CaCO_3_ and TOC MARs (Fig. 2b,c) are considered here as proxies for past productivity variations in response to changes in regional forcing like surface water hydrography and nutrient supply in the BoB.”

should read:

“CaCO_3_ and TOC MARs (Fig. 2b,c) are considered here as proxies for past productivity variations in response to changes in regional forcing like surface water hydrography and nutrient supply in the BoB. An earlier study by Phillips *et al*. has discussed possible influence of salinity/hydrographic changes on marine productivity indicated by CaCO_3_ MAR in the northern Bay of Bengal^1^.”

and:

“Overall diminished fresh water flux during colder and arid sub-stages caused thinning of low salinity cap leading to destabilization of the water column stratification which triggered enhanced nutrient entrainment by wind driven processes and convective mixing leading to enhanced productivity (enhanced CaCO_3_ MAR).”

should read:

“Overall diminished fresh water flux during colder and arid sub-stages caused thinning of low salinity cap leading to destabilization of the water column stratification which triggered enhanced nutrient entrainment by wind driven processes and convective mixing leading to enhanced productivity (enhanced CaCO_3_ MAR)^1^.”
