# Supplementary material for: Salinity stratification controlled productivity variation over 300 ky in the Bay of Bengal
Source: Sci Rep. 2017 Oct 31;7:14439. doi: 10.1038/s41598-017-14781-3 (PMC5663938; doi:10.1038/s41598-017-14781-3)
Supplement: Supplementary file 1 — Supplementary information [file 41598_2017_14781_MOESM1_ESM.pdf]

Supplementary information

Salinity stratification controlled productivity variation over 300 ky in the Bay of Bengal

R. Da Silva[1], A. Mazumdar[1*], T. Mapder[2], A. Peketi[1], R.K. Joshi[3], A. Shaji[4], P. Mahalakshmi[5], B. Sawant[1], B.G. Naik[1], M.A. Carvalho[1], S. K. Molletti[6]

[1]CSIR, National Institute of Oceanography, Goa;

[2]ACEMS, Queensland University of Technology,

[3]Geological Survey of India, Kolkata,

[4]Centre for Marine Living Resources & Ecology, Kerala,

[5]Flat No. CS-1, Block-C, Astral Garden, Panaji, Goa,

[6] Delta Studies Institute, Andhra University, Andhra Pradesh

*correspondence: maninda@nio.org

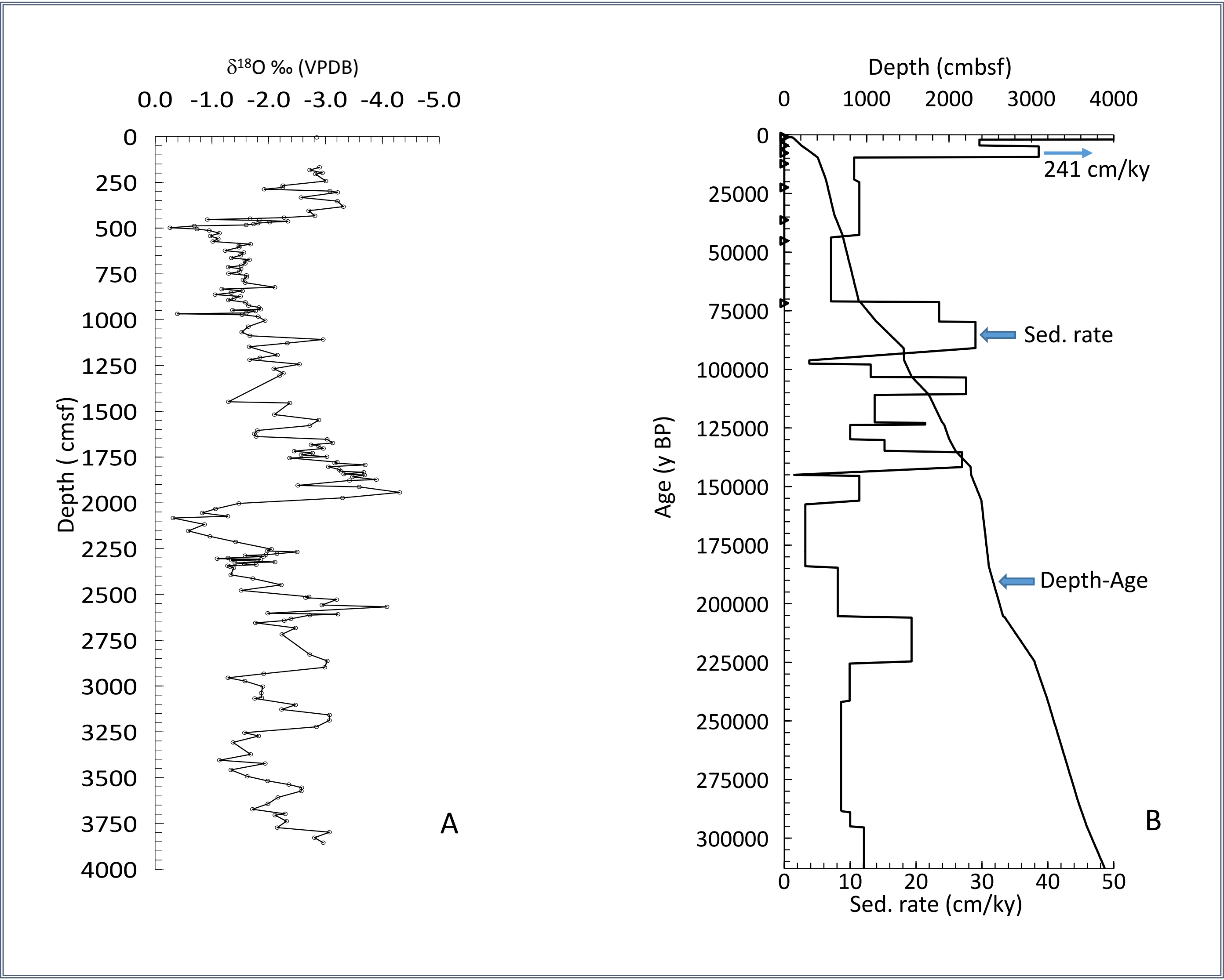

Supplementary Figure 1. a. $\delta^{18}O_{G.ruber}$ profile of MD161-19. b, Age-depth and model and sedimentation rate profile with calibrated radiocarbon ages (triangles) for MD161-19 (Ref. #12 in text). Age axis is established on the basis of $\delta^{18}O_{G.ruber}$ and calibrated radiocarbon ages.The sedimentation rate varies from 1.5 to 38.6 cm/ky except in the last 1114 yr where the sedimentation rate reaches peak value of 241 cm/ky

Statistical analyses

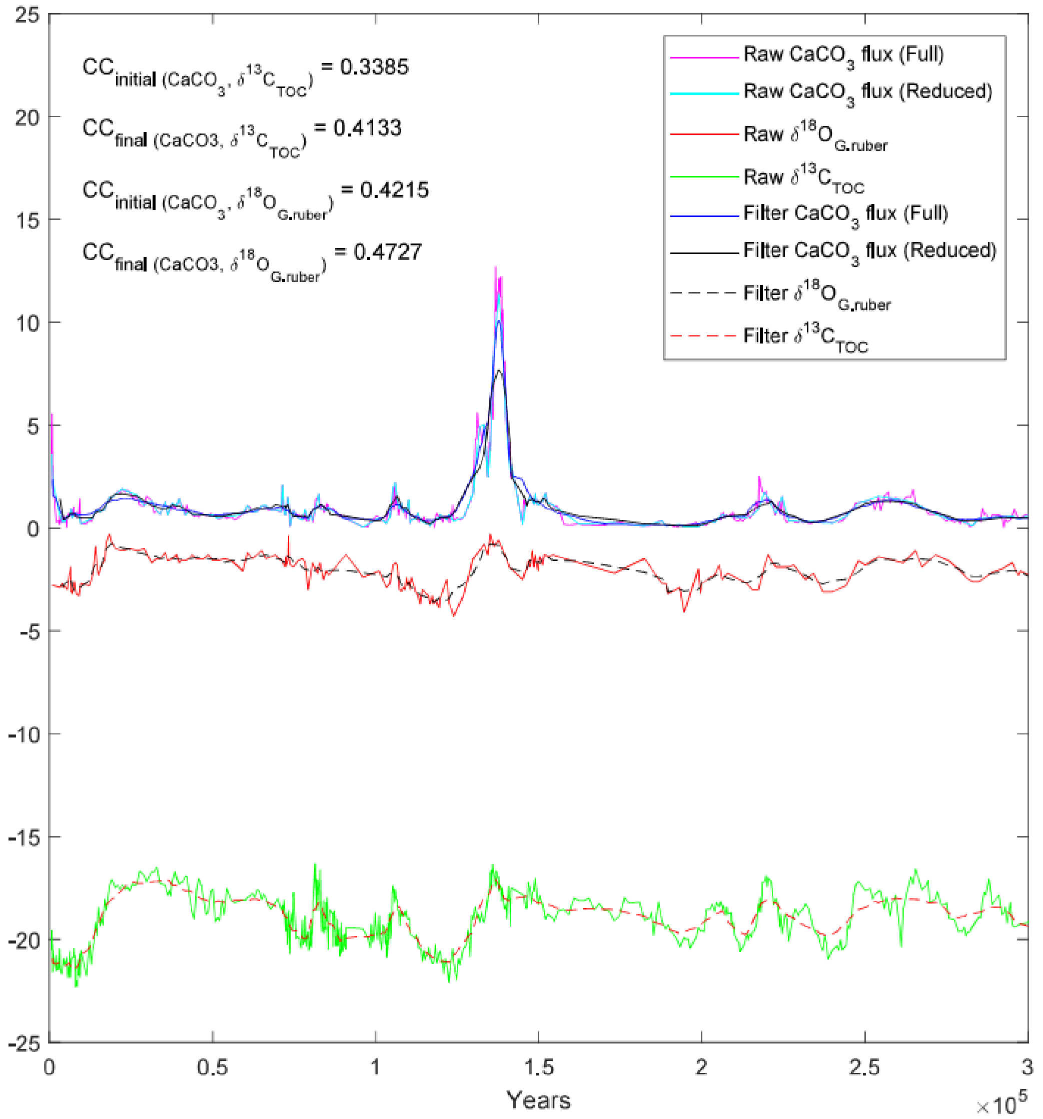

Supplementary Figure 2a: The raw data and the simple moving average (SMA) filtered data of the levels of $CaCO_3$ flux, $\delta^{18}O_{G.ruber}$, and $\delta^{13}C_{TOC}$ are plotted with respect to time in years. The four lines for $CaCO_3$ flux represent for raw full data (magenta, solid), raw truncated data (cyan, solid), filtered full data (blue, solid), and filtered truncated data (black, solid). Two lines in case of $\delta^{18}O_{G.ruber}$ stand for the raw data (red, solid) and the filtered data (black, dashed). The last two lines are the representative for $\delta^{13}C_{TOC}$ in raw (green, solid) and filtered data (red, dashed), respectively. The $CC_{initial}$ and CC_{final}, are the values of the respective Pearson's correlation coefficients between the species in subscript, calculated before and after applying the SMA filter.

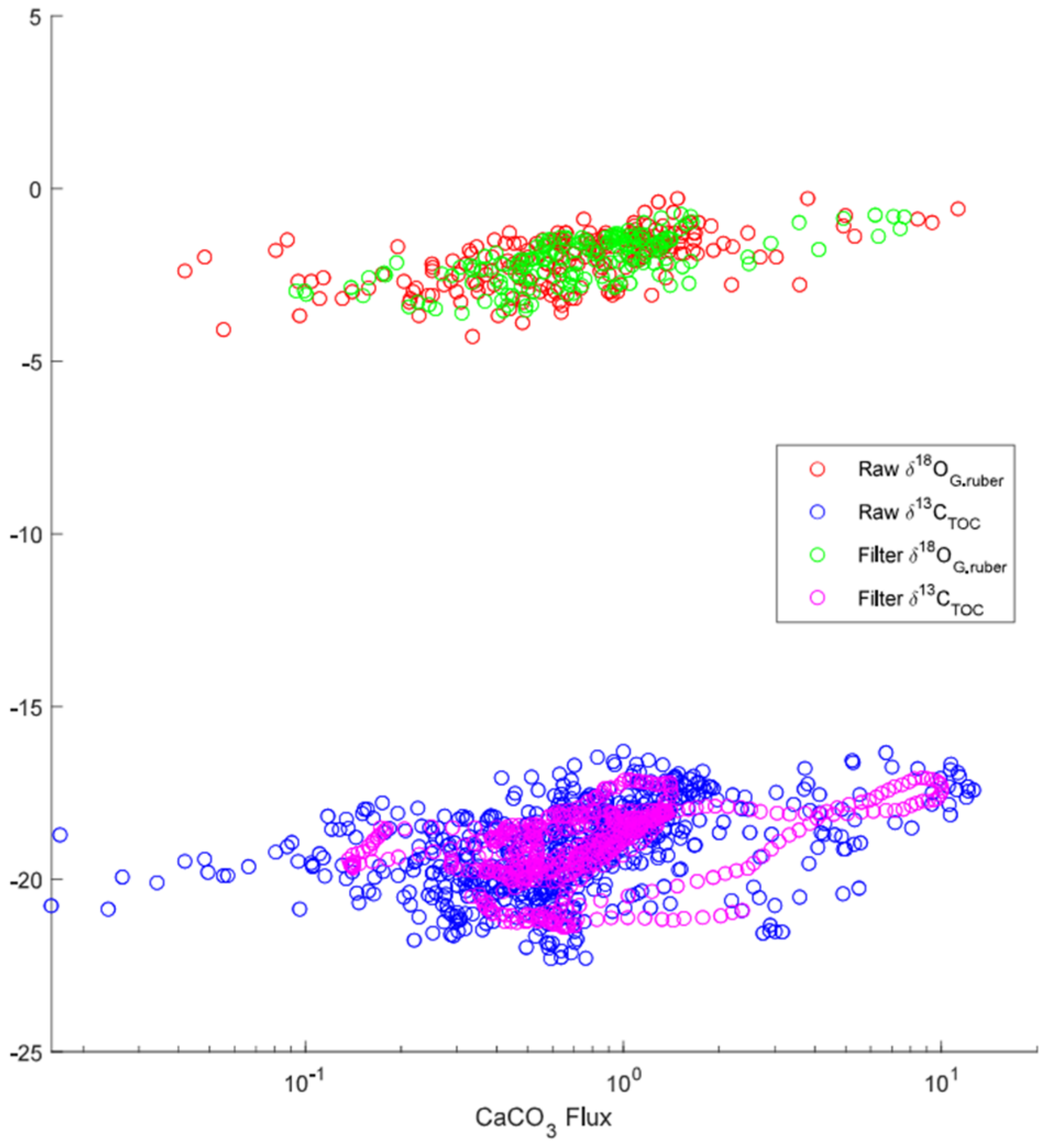

Supplementary Figure 2b: The raw and filtered data of $\delta^{18}O_{G.ruber}$, and $\delta^{13}C_{TOC}$ are plotted in scattered symbols with respect to level of $CaCO_3$ flux (raw and filtered, respectively). The red and green circles are depicting the raw and filtered $\delta^{18}O_{G.ruber}$ levels and the circles in blue and magenta represent for the same of $\delta^{13}C_{TOC}$. Please note the Log scale in $CaCO_3$ flux**.**

Supplementary Table-1

Sampling depth, callibrated age and oxygen isotope ratio of *Globigerinoides ruber* in core MD161-19. (Uploaded as excel file)

Supplementary Table-2

Sampling depths, calibrated ages, total inorganic carbon/organic carbon contents and marine, carbon stable isotope ratios of organic carbon, calculated paleo CO2aq, porosity, estimated dry bulk density and sedimentation rate measurements for MD161-19. (Uploaded as excel file.)
